# Immunological perspectives on atherosclerotic plaque formation and progression

**DOI:** 10.3389/fimmu.2024.1437821

**Published:** 2024-09-27

**Authors:** Hui Pi, Guangliang Wang, Yu Wang, Ming Zhang, Qin He, Xilong Zheng, Kai Yin, Guojun Zhao, Ting Jiang

**Affiliations:** ^1^ Affiliated Qingyuan Hospital, Guangzhou Medical University (Qingyuan People’s Hospital), Qingyuan, Guangdong, China; ^2^ Department of Microbiology and Immunology, Dali University, Dali, Yunnan, China; ^3^ Departments of Biochemistry and Molecular Biology and Physiology and Pharmacology, Cumming School of Medicine, University of Calgary, Calgary, AB, Canada; ^4^ Department of General Practice, The Fifth Affiliated Hospital of Southern Medical University, Guangzhou, Guangdong, China

**Keywords:** atherosclerosis, plaque evolution, immune response, immune cell heterogeneity, inflammatory microenvironment, immunotherapy

## Abstract

Atherosclerosis serves as the primary catalyst for numerous cardiovascular diseases. Growing evidence suggests that the immune response is involved in every stage of atherosclerotic plaque evolution. Rapid, but not specific, innate immune arms, including neutrophils, monocytes/macrophages, dendritic cells (DCs) and other innate immune cells, as well as pattern-recognition receptors and various inflammatory mediators, contribute to atherogenesis. The specific adaptive immune response, governed by T cells and B cells, antibodies, and immunomodulatory cytokines potently regulates disease activity and progression. In the inflammatory microenvironment, the heterogeneity of leukocyte subpopulations plays a very important regulatory role in plaque evolution. With advances in experimental techniques, the fine mechanisms of immune system involvement in atherosclerotic plaque evolution are becoming known. In this review, we examine the critical immune responses involved in atherosclerotic plaque evolution, in particular, looking at atherosclerosis from the perspective of evolutionary immunobiology. A comprehensive understanding of the interplay between plaque evolution and plaque immunity provides clues for strategically combating atherosclerosis.

## 1 Introduction

Atherosclerotic cardiovascular disease (ACD) is a prominent global cause of mortality (1). Atherosclerosis is a slow process, characterized by multifocal structural changes in the vascular wall of large and medium- sized arteries, which result in the development of atherosclerotic plaques (2). Atherosclerotic plaques are the pathophysiological basis of almost all arterial vascular diseases. Advanced plaques may rupture, triggering thrombosis that blocks arteries and disrupts blood flow, leading to an array of life-threatening clinical outcomes called major adverse cardiovascular events (MACEs) (3). Past epidemiologic studies have revealed many risk factors for atherosclerosis, among which traditional risk factors include dyslipidemia, hypertension, hyperhomocysteinemia, hyperfibrinogenemia, diabetes mellitus, smoking, obesity, and genetic predisposition. In recent years, non-traditional drivers such as sleep disorders, lack of exercise, air pollution, environmental stress, as well as inflammation and clonal hematopoiesis associated with the immune system have also received attention (3, 4).

The evolution of atherosclerotic plaques can be broadly categorized into three stages: initiation, progression and complications. It has been shown that fatty streaks are the initial marker of atherosclerosis, which develops in four steps: low-density lipoprotein (LDL) cholesterol uptake, endothelial cell (EC) activation, leukocyte activation and foam cell formation (5). During the development of fibrous plaques, atherosclerotic plaques experience a shift from fatty streaks to intimal growth, and this step is marked by the formation of a lipid-rich necrotic core covered by a fibrous cap. The fibrous cap is composed of vascular smooth muscle cells (VSMCs) which migrate to the side of the arterial lumen and VSMC-derived extracellular matrix (ECM). The atherosclerotic plaques can rupture at the point where the fibrous cap is thinnest exposing the material inside to blood tissue triggering thrombosis. If the lumen is blocked, the narrowing of the diseased artery can also lead to other complications such as heart and brain infarction (6, 7).

Among the many factors that influence the evolution of atherosclerotic plaques, the immune system plays a significant role. During the formation and development of atherosclerotic plaques, the local microenvironment undergoes a series of complex changes, accompanied by the infiltration of multiple immune cells. Evidence suggests that circulating monocytes and resident vascular macrophages are the earliest immune cells recruited into early atherosclerotic plaques (8). Following this, various immune cells such as neutrophils, natural killer (NK) cells, DCs, T cells and B cells gradually infiltrate the plaque and perform their regulatory functions. Subpopulations of leukocytes in the arterial wall are heterogeneous they play a pro-inflammatory or regulatory role in atherosclerotic plaque formation. Most patients with atherosclerosis are immunocompetent individuals (9). The key takeaway about the impact of immune function is that it is complex, and immunomodulation can positively or negatively affect the evolution of atherosclerotic plaques.

The molecular signals that regulate leukocyte recruitment to atherosclerotic lesion sites are complex, and chemokines and their receptors play a key role and have received much attention in the study of atherosclerosis. Chemokines are a class of small, secreted cytokines with chemotactic properties. Depending on the location of their cysteine residues, they can be divided into four subclasses: C, CC, CXC, and CX_3_C. They act mainly by binding to specific G-protein-coupled chemokine receptors (10). In atherosclerotic lesions, chemokines and many of their receptors are expressed in endothelial cells, leukocytes, and smooth muscle cells, with highest expression especially in regions near the necrotic core. They are widely involved in all stages of atherosclerosis by promoting immune cell adhesion, migration, infiltration, differentiation, and homing to the lesion site (11).

With the advent and advancement of various experimental techniques, we have gained an understanding of the mechanisms involving the immune system in plaque evolution. Here, we view atherosclerotic plaque lesions from the perspective of evolutionary immunobiology, specifically reviewing the immunologic aspects of plaque evolution, including the immune responses involved, the effects of immune cell heterogeneity, and plaque dynamics in the inflammatory microenvironment. The goal is to provide insight into the search for immunotherapy for atherosclerosis.

## 2 Understanding atherosclerotic plaque evolution

In 1859, Charles Darwin coined the phrase “natural selection” to describe the process of evolution as observed by the different survival outcomes of individuals under environmental stress owing to phenotypic differences. Natural selection has provided a crucial force in evolution (12). It also happens in the evolution of atherosclerotic plaques, as the same mechanism applies to immune cells infiltrating the plaque. The host immune system is a major source of selection pressure during the evolution of atherosclerotic plaques. The immune system has different cell types, states, and positions. The complex networks, interactions and reactions of immune cells give rise to a cellular ecosystem consisting of numerous cell types accompanied by the genetic diversity of antigen receptors (13). Under selective pressure from the immune system, immune cells exhibit differential phenotypes that distinctively influence the evolution of plaques.

Many believe that atherosclerosis is an inevitable progressive process that develops over time, but current evidence supports a more dynamic and discontinuous evolution of the atherosclerotic plaques (14, 15). Fatty streaks are the earliest form of atherosclerotic plaque lesions. Regardless of the prevalence of coronary artery disease, fatty streaks are observed in the aorta of children in all countries. These early pathologies may progress to more advanced lesions or recede. The fatty streaks progress to fibrous plaques, occurring late in the second decade of life and early in the third decade. Atherosclerotic plaque regression is unlikely to occur during this phase. The progression of fibrous plaques is associated with various complications: calcification, internal bleeding, ulceration, and the release of embolic fragments, as well as the formation of blot clots. Thrombosis causes acute events like myocardial infarction and ischemic stroke (4, 6, 7). Generally, plaques take decades to form and do not develop consistently or steadily. Moreover, once fibrous plaques develop, they are more prone to a variety of complications, which can be life-threatening in severe cases.

## 3 Immune responses in atherosclerotic plaques

**Figure 1:**
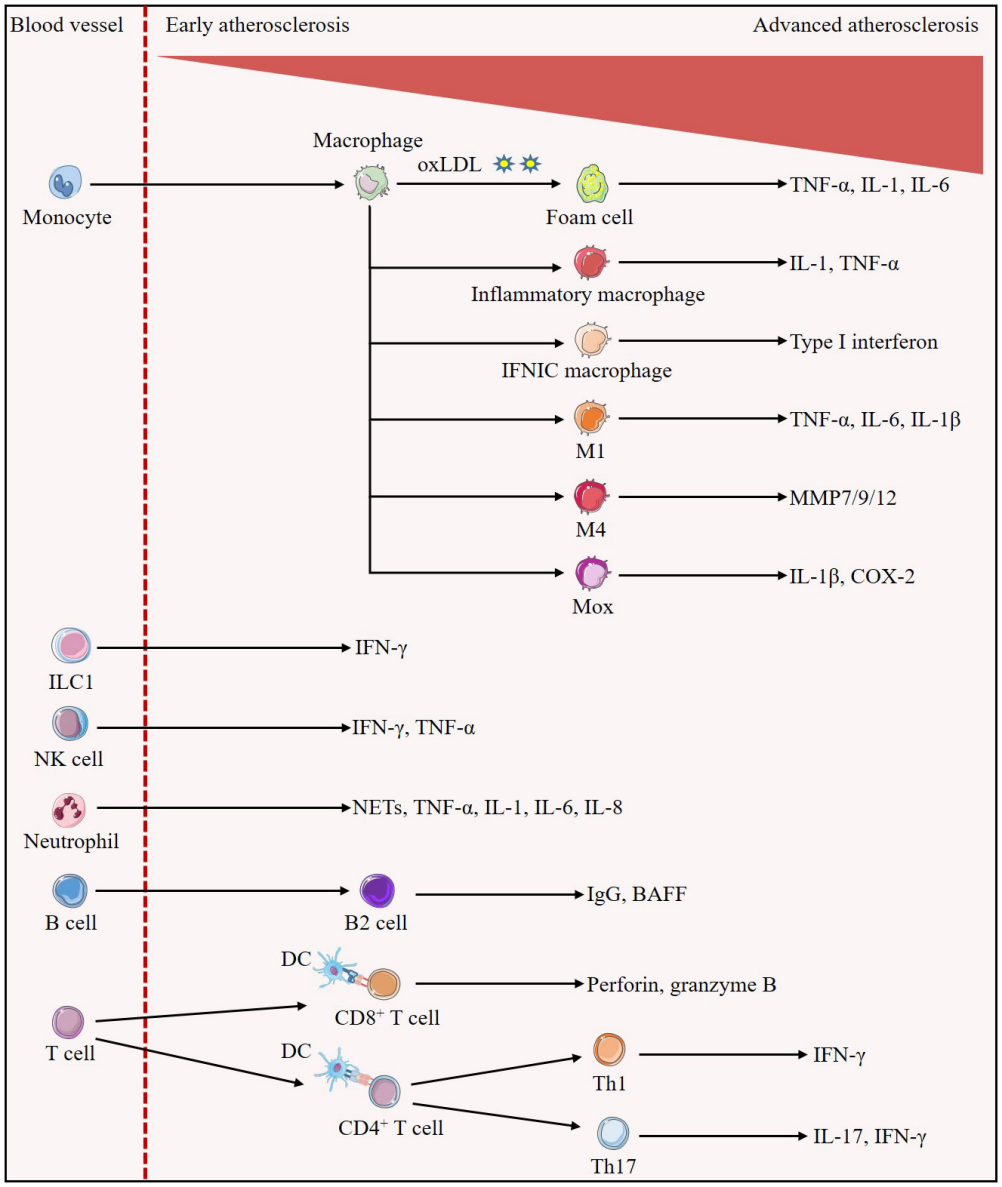
Evolution of immune cells in the development of atherosclerotic plaques. Early in the lesion, monocytes/macrophages predominate. Monocytes differentiate into macrophages, which can uptake oxLDL to become foam cells, and also differentiate into inflammatory macrophages and IFNICs macrophages, which secrete IL-1, TNF-α, and type I interferon to exert pro-inflammatory effects, respectively; in addition, they can also be polarized into the M1, M4, and Mox phenotypes, which secrete pro-inflammatory cytokines such as IL-1β, TNF-α, IL-6, or MMP, promoting atherosclerosis. In all stages of atherosclerosis, ILC1 is identified as the most abundant subset of ILCs and is involved in reinforcing plaque formation through the production of IFN-γ. Atherosclerotic lesions trigger migration and activation of NK cells as well as release of pro-atherogenic factors IFN-γ and TNF-α. Neutrophils secrete pro-inflammatory mediators such as NETs, TNF-α, IL-1, IL-6 and IL-8, increasing plaque inflammation and susceptibility to rupture. A large number of B cells with a structure similar to tertiary lymphoid organs are found in the adventitial layer of blood vessels, in which B2 cells secrete mediators that aggravate atherosclerosis such as IgG and BAFF. In advanced atherosclerosis, numerous T cells homing to the plaque. Activated DCs present cognate peptides on MHC class I molecules to CD8^+^T cells and on MHC class II molecules to CD4^+^T cells. Activated CD8^+^T cells exert atherogenic effects by secreting perforin and granzyme B and inducing cell death. CD4^+^T cells are polarized into different subsets, and Th1 cells are able to further stimulate foam cell formation and exacerbate atherosclerosis through the secretion of their major cytokine, IFN-γ. IL-17 secreted by Th17 cells may accompany the increase of IFN-γ to promote plaque inflammatory response and play pro-atherogenic roles.

The immune system is categorized into innate and adaptive responses. As the first line of defense against invading pathogens, innate immunity is characterized by its ability to produce rapid and nonspecific responses. The main types of cells in the innate immune system involve neutrophils, monocytes/macrophages and DCs (16). Innate immune cells are capable of recognizing pathogen-associated and damage-associated molecular patterns by means of pattern recognition receptors, encompassing scavenger receptors (SRs) and toll-like receptors (TLRs) (17). Adaptive immunity is more specific, but slower. Adaptive immunity is mainly composed of T cells and B cells that identify the specific epitopes on pathogens as well as antigens for which they produce a variety of antigen-specific T cell receptors and immunoglobulins (18). The evolution of immune cells plays a key role in the development of atherosclerotic plaques, actively promoting plaque formation and development (**Figure 1**).

### 3.1 The arterial wall

The arterial wall is composed of three main layers: the intima, the media, and the adventitia. The intima is the innermost layer in contact with blood flow and is covered by a single layer of ECs on one side of the lumen (vascular endothelium) and an inner elastic layer on the peripheral side. The media consists mainly of VSMCs surrounded by ECM. The adventitia is composed of fibroblasts and connective tissue (19). The vascular endothelium is the initial obstacle for circulating molecules, cells, or pathogens in the bloodstream (20). Disruption of intravascular homeostatic regulatory mechanisms leads to endothelial dysfunction (21), which facilitates the development and progression of atherosclerotic plaques and can be considered as an independent vascular danger factor.

In addition to endothelial dysfunction, perivascular adipose tissue (PVAT) dysfunction is also a key factor in plaque formation. PVAT, a mixture of brown and white adipose tissue that surrounds most of the vascular system, is thought to be an active component of the vascular wall that regulates vascular homeostasis and influences the pathogenesis of atherosclerosis (22). Under physiological conditions, PVAT exhibits potent anti-atherosclerotic properties through its thermogenic capacity and secretion of several bioactive molecules such as adiponectin and NO. Under the influence of pathological states, such as obesity, diabetes and hypertension, which are metabolic diseases, PVAT becomes dysfunctional and secretes high levels of pro-inflammatory factors such as leptin, resistin, inflammatory cytokines tumor necrosis factor alpha (TNF-α) and interleukin (IL) -6, and chemokines such as monocyte chemotactic protein-1 (MCP-1), which induce endothelial dysfunction, immune cell infiltration and inflammation, and ultimately promote the development of atherosclerosis (23, 24).

Atherosclerotic plaque formation begins with endothelial dysfunction followed by LDL accumulation and its modification in the intima (25). As a result of impaired endothelial barrier function and elevated blood levels of plasma lipids, LDL infiltrates the endothelium and deposits on the arterial wall via endocytosis and is retained in the intima by ECM macromolecules (26). As soon as they enter the subendothelial space, the captured LDL particles are oxidatively modified to activate ECs and VSMCs. Activated ECs induce selective monocytes to be recruited to the intima, rapidly differentiating into tissue macrophages and transforming into foam cells through accumulation of trapped and modified lipoproteins. Notably, a small proportion of foam cells are derived from VSMCs and ECs (27, 28). The accumulation of foam cells is a driving factor in plaque growth. Overall, the combination of endothelial cell injury, oxidative modification of LDL, and macrophage activation lead to increased LDL uptake, which promotes the formation and progression of atherosclerosis.

### 3.2 Innate immunity

Lipid accumulation stimulates the production of inflammatory mediators and cytokines by ECs. EC activation triggers the release of leukocyte adhesion molecules (AMs) and chemokines that stimulate inflammatory monocyte migration and infiltration. Infiltrated monocytes in the intima are stimulated to mature into macrophages by macrophage colony-stimulating factor (M-CSF) and granulocyte-macrophage colony-stimulating factor (GM-CSF) produced by activated ECs (29). Macrophages express a range of SRs, including CD36, CD68, lectin-type oxidized LDL receptor 1 (LOX-1), SR-A, and SR-B1, which mediate ligand internalization, degradation, and antigen presentation. In contrast, TLRs can directly induce an inflammatory response (17). Many TLR family members such as TLR1, TLR2, TLR4, and TLR5 that are detected in human plaque and atherosclerosis mouse models are mainly expressed by macrophages and ECs (30). Of these, TLR4 is noteworthy as it is highly expressed in human atherosclerotic plaques and its expression is upregulated by oxidized LDLs (oxLDLs) (31). Myeloid differentiation factor 88 (MyD88) is a critical adaptor protein involved in TLR4 signaling. Apolipoprotein E -deficient (*ApoE^-/-^*) mice lacking both TLR4 or MyD88 show reduced atherosclerosis (32). Overall, SRs and TLRs play a significant role in the evolution of atherosclerotic plaques.

Other innate immune cells such as neutrophils, Innate lymphoid cells (ILCs) and DCs can also influence the evolution of atherosclerotic plaques. Neutrophils are the major responders to tissue damage and infection, releasing pro-inflammatory mediators to neutralize dangers such as toxins (33). Neutrophils aggravate endothelial dysfunction, induce monocytes to move into atherosclerotic lesions, activate macrophages and promote foam cell formation by releasing granule proteins. Analysis of human plaque specimens suggests that neutrophil-derived proteases and reactive oxygen species (ROS) contribute to plaque destabilization (34). In addition, cholesterol crystals trigger neutrophils to release neutrophil extracellular traps (NETs), complex structures composed of nuclear chromatin and proteins sourced from the nucleus, granules, and cytoplasm. NETs cause macrophages to release cytokines that activate Th17 cells, expanding the recruitment of immune cells in atherosclerotic plaques (35, 36). Therefore, neutrophils play a facilitating role in the evolution of plaques.

ILCs are a unique type of lymphocytes that have been newly discovered in recent years. Five distinct subsets have been identified: ILC1s, ILC2s, ILC3s, lymphoid tissue-inducing cells and NK cells. ILCs are mainly localized in barrier tissues such as skin, intestinal mucosa, and lung, and play an important role in innate immune response to infections, lymphangiogenesis, and remodeling of damaged tissues by responding to microenvironmental signals released from surrounding tissue (37, 38). ILC1s are the predominant subset of ILCs in mouse and human atherosclerotic plaques and are involved in enhancing plaque formation by producing IFN-γ in response to stimulation by the transcription factor T-bet; conversely, removal of ILC1 attenuated plaque formation in *ApoE^-/-^* mice (39). ILC2s require the transcription factor GATA3 and are protective against atherosclerosis through the production of type 2 cytokines that promote B1 cell proliferation and IgM production as well as modulate diseased macrophages (40). ILC3s are the least abundant lymphocyte subset in atherosclerosis, and its dependence on the transcription factor RORγt for the production of IL-17 and IL-22 on the effects of atherosclerosis has been inconsistently investigated, leading to our lack of clarity on the role of ILC3s in atherosclerosis (41). Because of the overlap in gene expression between ILCs and T cells and the limitations of high-dimensional single-cell techniques, our information on the role of ILCs in atherosclerosis is limited, and the exact mechanisms require more research.

NK cells are innate immune system effector lymphocytes that sense pathologic alterations or stressed cells by activating receptors and play a cell-mediated cytotoxic role through the release of granzymes and perforin or cytokines, as in the case of interferon-gamma (IFN-γ) (42). NK cells have been detected in both human and mouse atherosclerotic plaque lesions (43, 44). Nevertheless, there is some controversy regarding the role of NK cells in atherosclerotic plaques. An early study of the role of NK cells in atherosclerosis using beige LDL receptor^-/-^ (*Ldlr^-/-^*) mice showed that NK cells have atheroprotective effects (45). Another study investigating the impact of NK cell loss and gain of function in atherosclerosis development demonstrated that NK cells possess a pro-atherogenic role. Their production of interferon-γ, perforin, and granzyme B, as well as the expansion of the necrotic core within the lesion, facilitates the evolution of atherosclerotic plaques (46). Therefore, additional research is necessary to elucidate the precise role of NK cells in atherosclerotic plaques.

DCs are present in healthy arteries and can accumulate in atherosclerotic lesions. They are especially enriched in rupture-prone areas within atherosclerotic plaques, which can lead to plaque destabilization, and are involved in a variety of pathogenic and protective mechanisms during plaque formation. In the earliest stages of plaque formation, DCs may uptake lipids and form a foam-cell-like appearance, as well as regulate lipid metabolism through other mechanisms (47). DCs can also produce many pro-inflammatory cytokines, including TNF, IL-6, and IL-12, which are all pro-atherosclerotic (48). In addition, DCs in the arterial wall also express TLR4, which performs an essential role during the formation and development of atherosclerotic plaques. DCs as specialized antigen-presenting cells link innate and adaptive immunity by presenting antigens to T cells. Thereby, DCs significantly influence the occurrence and advancement of atherosclerotic lesions.

### 3.3 Adaptive immunity

The antigen-specific adaptive immune responses are triggered after the innate immune response and are mediated by T and B cells (49). The hypothesis that adaptive immunity plays a role in human atherosclerosis has been long studied.

T cells are recruited into atherosclerotic plaques through a mechanism that resembles monocyte recruitment via the receptor C-X-C motif chemokine receptor 3 (CXCR3), which is expressed by all CD4^+^ T lymphocytes in the same lesion (50). By inhibiting CXCR3 and blocking the migration of T cells from the circulation into the atherosclerotic plaque, plaque formation can be reduced (51). In addition to CXCR3, other chemokine receptors have been found to be responsible for T cell recruitment. Galkina et al. (52) found that CXCR6 expressed on multiple T cell subsets can facilitate T cell aggregation in the aorta and thus promote the development of atherosclerosis. Li et al. (53) demonstrated that CD4^+^ T cells homing to atherosclerotic plaques specifically required CC chemokine receptor 5 (CCR5), and blocking or knocking down CCR5 significantly prevented T cell recruitment. Similarly, CCR2 deficiency limits T cells entering the arterial intima and suppresses atherosclerosis (54). Recent studies have found that deletion of CCR7 not only leads to reduced plaque content in mice, but also results in disturbed access of T cells to and from the site of plaque inflammation (55).The *ApoE^-/-^* mice lacking CD4^+^ T cells show reduced atherosclerosis (56). CD4^+^ T cells from *ApoE^-/-^* mice were transferred to *ApoE^-/-^* mice crossed with severe combined immunodeficiency (SCID) strains of mice, leading to greatly increased fatty streak lesions compared to immunocompetent *ApoE^-/-^* mice (57). These studies indicate that CD4^+^ T cells play a pro-atherogenic role in the early stages of atherosclerotic plaque formation.

The CD4^+^ T cells that play a pro-atherosclerotic role are mainly T helper (Th) cells, especially Th1 cells, while regulatory T cells (Tregs) have been identified to play an anti-atherosclerotic role. CCL1 is a potent leukocyte chemotactic agent that plays an essential role in recruiting Tregs by binding to CCR8. Disruption of the CCL1-CCR8 axis facilitates atherosclerosis by inhibiting IL-10 production and Tregs recruitment and function (58). Likewise, CCL17 acts as a chemoattractant for CCR4^+^ T cells that inhibits Tregs migration and expansion, further limiting their function. Blocking CCL17 increases Tregs infiltration and reduces atherosclerosis (59). In addition, a study by Shao et al. (60) found that in *ApoE^-/-^* mice, CCR5 mediated Tregs homing to the aorta in the presence of IL-35, which ultimately inhibited atherosclerosis by maintaining the suppressive functions of Tregs.

Various studies have come to distinct conclusions about the effects of CD8^+^ T cells on atherosclerosis. In early human atherosclerotic plaques, CD8^+^ cytotoxic T cells (CTLs) are not as plentiful as CD4^+^ T cells, but in late human plaques they represent up to 50% of the leukocytes. CTLs are activated during hypercholesterolemia and can facilitate plaque inflammation and instability in lesions (61). Mice lacking antigenic peptide transporter 1 (TAP1) have lower numbers of CD8^+^ T cells and *ApoE^-/-^Tap1^-/-^* mice exhibit the same atherosclerotic lesions, immune cell infiltration and lipid accumulation as *ApoE^-/-^* mice (62). This indicates that CD8^+^ T cells have no or minimal effects on atherosclerotic plaques. However, other studies using the transfer of CD8^+^ T cells lacking perforin, granzyme B or TNF-α have shown that CD8^+^ T cells contribute to the evolution of atherosclerotic plaques by perforin- and granzyme B-mediated cytotoxicity and TNF-α-mediated inflammation by promoting apoptosis in macrophages, ECs and VSMCs and the development of the necrotic core (63). Thus, CD8^+^ T cells may play a secondary role in the early stages of atherosclerotic plaque evolution, but they may be triggered by intracellular infection, which then promotes the accumulation of atherosclerotic plaque or regulates the later stages of atherosclerosis.

Atherosclerotic plaque formation is intensely influenced by diverse arms of the immune system, including B lymphocytes (64). B cells are detected in atherosclerotic lesions, which have a structural organization similar to tertiary lymphoid organs and contribute to atherosclerotic plaque formation (65). Two major subsets of B cells have been found in atherosclerotic plaques. B1 cells spontaneously produce natural IgM antibodies to protect against atherosclerosis. Conventional B2 lymphocytes produce pro-atherogenic IgG, IgA, and IgE antibodies (64). In fact, it was discovered 40 years ago that atherosclerotic plaques contain immunoglobulins, particularly IgM and IgG, which are present in all stages of lesion development (66, 67). In addition, specific monoclonal antibodies against different epitopes of oxLDL, now referred to as oxidation-specific epitopes, have been discovered both in human and mouse plasma and in atherosclerotic plaques (68). B cells regulate plaque evolution by releasing antigen-specific antibodies that mediate humoral immune responses.

The earliest evidence that B cells are involved in atherosclerosis in mice came from Caligiuri et al. (69) who indicated that increased atherosclerosis in *ApoE^-/-^* mice after splenectomy was reversed through adoptive transfer of splenocytes, supporting an atheroprotective role. In addition, Major et al. (70) demonstrated that transplantation of B-cell-deficient mouse bone marrow to *Ldlr^-/-^* mice caused an increase in atherosclerotic plaque formation, clearly demonstrating the protective effect of B cells against atherosclerotic lesions. Further, injection of polyclonal immunoglobulin preparations into *ApoE^-/-^* mice resulted in a reduction in fatty streaks and fibrous plaques, indicating that atherosclerosis could be inhibited (71). Consistent with these results, the use of bone marrow transplants, whereby *Ldlr^-/-^* mice were reconstituted with wild-type or IL-5^-/-^ bone marrow, resulted in reduced secretion of T15/EO6 clonotype natural IgM antibodies and accelerated atherosclerosis (72). The above studies suggest that humoral immunity and B cells have properties that inhibit the evolution of atherosclerotic plaques. In contrast, the use of CD20-specific monoclonal antibody-mediated depletion of mature B cells reduced the size of atherosclerotic plaque lesions in *ApoE^-/-^* and *Ldlr^-/-^* mice (73). Furthermore, relay transfer of conventional B2 cells into lymphocyte-deficient *ApoE^-/-^* mice and B cell-deficient *ApoE^-/-^* mice effectively promoted the development of atherosclerotic plaques (74). These studies, in turn, show that B cells have a pro-atherosclerotic role. Overall B cells have both a protective and pathogenic role in atherosclerosis depending on the subclass, but more research is required to elucidate the role of each subclass.

## 4 Evolutionary mechanism for untreated atherosclerotic plaque under immunomodulation

Atherosclerosis is now recognized as a chronic inflammatory disease, with inflammation having an essential role in all stages of the pathogenic process, encompassing plaque formation, progression, and rupture. Inflammation is the response to the existence of exogenous and endogenous antigens by the immune system. In general, driven by the immune system, inflammation profoundly influences the evolutionary trajectory of atherosclerotic plaques.

### 4.1 Inflammation is the driving factor of atherosclerotic plaque initiation

Inflammation underlies atherosclerotic plaque formation. Plaque inflammation is driven by cytokines, chemokines, AMs, inflammatory signaling pathways, bioactive lipids, and immune cells (75, 76). Nearly all conventional risk factors for atherosclerosis are associated with and actively contribute to inflammatory processes.

Infiltration and retention of oxLDL in the arterial wall serve as critical initiating events that trigger the inflammatory response and facilitate atherosclerotic plaque formation (77). It has been demonstrated that atherosclerotic plaques in both humans and experimental animals contain oxidatively modified LDL (78). OxLDLs contain oxidized lipids, and the products of their degradation make them critical inflammatory components that contribute to the development of atherosclerotic plaques. According to the level of LDL oxidation, oxLDLs are divided into minimally modified LDL (mmLDL) or extensively oxLDL (79). The extensively oxLDL and mmLDL can trigger pro-inflammatory responses in ECs and macrophages, leading to endothelial dysfunction and recruitment of leukocytes to the site of the lesion. Furthermore, mmLDL induces upregulation of TLR2 and TLR4 expression in monocytes/macrophages, and increased TNF-α secretion promotes plaque inflammatory responses (80). In addition, oxLDL and corresponding antibodies bind to form immune complexes (ICs) that have pro-atherogenic and pro-inflammatory properties and ICs activate the inflammasome through signaling at multiple receptors (81, 82). NOD-like receptor family pyrin domain-containing protein 3 (NLRP3) is the most broadly studied of the numerous inflammasomes and an influential regulator in the pathogenesis of cardiovascular disease. A characteristic feature of atherosclerotic plaques is increased expression of the NLRP3 inflammasome component (83), and inhibition of the NLRP3 inflammasome using MCC950 inhibitor reduces the development of atherosclerotic plaques in *ApoE^-/-^* mice (84). In conclusion, oxLDL promotes plaque inflammatory responses by interacting with immune components.

SRs are distributed on arterial vessel wall cells and vascular ECs, and oxLDL binds to the corresponding receptors to promote the evolution of atherosclerotic plaques through multiple mechanisms. CD36 is a pattern recognition receptor expressed on multiple cell types and is a part of the class B family of SRs. CD36 is not only involved in the uptake of oxLDL and foam cell formation, but also in atherosclerotic plaque formation by interacting with oxLDL and triggering an inflammatory response (85). LOX-1 belongs to the C-type hemagglutinin family and is a specific receptor for oxLDL. LOX-1 is expressed by ECs, macrophages and VSMCs in various stages of atherosclerotic lesions (86). Overexpression of LOX-1 facilitates endothelial dysfunction, vascular inflammation, and plaque formation, and participates in the destabilization of atherosclerotic plaques *in vivo (* 87, 88). In contrast, LOX-1 deficiency maintains endothelial function and reduces atherosclerosis development (89). Altogether, oxLDL recognizes the corresponding receptors and promotes the development of plaque inflammation.

### 4.2 Inflammation accelerates the evolution of atherosclerotic plaque

The accumulation of oxLDL and its binding to receptors initiate plaque inflammation, while the infiltration of immune cells, the activation of plaque-associated inflammatory signaling pathways, and the response to host tissues and microorganisms maintain the development of inflammation, thereby further accelerating the evolution of plaque.

Infiltration and accumulation of pro-inflammatory and anti-inflammatory leukocytes in the intima of the arterial wall are hallmarks of atherosclerotic plaque formation and progression, as well as drivers of atherosclerotic lesion growth. Immune cell infiltration is triggered by chemokines and AMs (90). Members of the chemokine and AM families mediate the recruitment of immune cells to infiltrate the lesion and accelerate the development of the plaque inflammatory response. In addition, they modulate cellular homeostasis, leading to endothelial dysfunction as well as involvement in thrombosis (91, 92). Many previous studies have focused on P-selectin glycoprotein ligand-1 (PSGL-1), an adhesion ligand which is expressed on leukocytes and ECs. Knockdown of PSGL-1 in an *ApoE^-/-^* mouse model reduced monocyte infiltration and leukocyte adhesion, decreased atherosclerotic plaque area and was protective against atherosclerosis (93, 94). Furthermore, systemic low-grade chronic inflammation triggered by the common risk factor obesity is also capable of promoting immune cells infiltration, including lymphocytes and macrophages, which impairs systemic metabolism by exacerbating adipose tissue inflammation, leading to cholesterol deposition in the blood vascular wall, with the ensuing immune process supporting the growth of atherosclerotic plaques and contributing to cardiovascular complications (95).Overall, the development and growth of plaques in atherosclerosis cannot occur without the infiltration of immune cells.

Signaling pathways that are mediated by immune, inflammatory mediators are associated with the evolution of atherosclerotic plaques. Nuclear factor-κB (NF-κB) serves as the primary transcription factor in the inflammatory response. The NF-κB pathway is induced and activated by pro-inflammatory cytokines, adhesion molecules, chemokines and growth factors and exerts a pivotal role in atherosclerotic plaque inflammation (96). Activated NF-κB is observed in several cells including macrophages, VSMCs and ECs in atherosclerotic lesions. Studies have shown that activated NF-κB is involved in many features of atherosclerosis, including mediating foam cell formation, enhancing vascular inflammation, stimulating VSMCs proliferation and migration, exacerbating vascular calcification, promoting plaque formation and destruction, and regulating vascular apoptosis (97). In *Ldlr^-/-^* mice fed an atherogenic diet for a prolonged period, the NF-κB signaling pathway in the endothelium was activated, leading to increased atherosclerotic plaque formation (98). Furthermore, TLRs are expressed in all atherosclerotic plaque immune cells and are engaged in the inflammatory response to plaques. The TLRs signaling pathway can be broadly divided into MyD88-dependent and non-MyD88-dependent pathways. MyD88 activates NF-κB and MAPK signaling pathways and attracts the expression of inflammatory cytokines. The non-MyD88-dependent pathway, TIR-domain-containing adapter protein-inducible interferon (IFN)-β (TRIF)-dependent pathway, activates IFN regulatory factor 3 (IRF3) and induces the expression of type I IFNs (99, 100). Among these, TLR4 signaling plays an important role in activating atherosclerotic plaque inflammation and lipid accumulation. Knockdown of Adenosine triphosphate-binding cassette transport increases TLR4 and MyD88/TRIF signaling in macrophages, enhances expression of inflammatory cytokines and accelerates plaque inflammation (101). In general, TLRs can induce the synthesis of pro-inflammatory mediators as well as promote plaque inflammation through activating complex cell signaling pathways. Next, janus kinase (JAK)-signal transducer and activator of transcription (STAT) signaling pathways exert pro-inflammatory effects in atherosclerotic plaques by inducing diverse pro-inflammatory cytokines such as IL-6, TNF-α and IL-1 (102). The JAK/STAT pathway is activated when various cytokine receptors on the cell membrane bind to the corresponding ligands. The JAK family is composed of four members: JAK1, JAK2, JAK3, and TYR2, while the STAT family is composed of seven members: STAT1, STAT2, STAT3, STAT4, STAT5a, STAT5b, and STAT6. Gharavi et al. (103) found that activated STAT3 was predominantly present in ECs and inflammatory cells in inflammatory areas of atherosclerotic plaques, with less in non-inflammatory areas. Mice with STAT3 knockout in the endothelium showed reduced fatty streak formation and decreased macrophage content compared to wild-type mice. In addition, there is growing evidence that the Wnt pathway is involved in multiple processes in the evolution of atherosclerotic plaques, including regulation of endothelial dysfunction, VSMC proliferation and migration, regulation of inflammation and foam cell formation, and pathological angiogenesis and calcification, all of which are key processes in plaque formation and stabilization (104). In conclusion, in the plaque inflammation microenvironment, crosstalk between signaling pathways further accelerates inflammation and plays a role in plaque evolution.

Research into atherosclerotic plaque development has also focused on the host tissues and microorganisms that may influence this process. Endothelial-mesenchymal transition (EndMT) is present in atherosclerotic plaques, where ECs can obtain a mesenchymal phenotype, with loss of expression of EC markers and function, and gain of expression of mesenchymal cell markers and function, directly contributing to plaque evolution (105). In addition to this, the brown adipocyte-specific PPARγ knockout mice were crossed with *ApoE^-/-^* mice and the resulting double knockout showed increased vascular and systemic inflammation and significantly increased atherosclerotic lesions (106). Thus, host cells and tissues can contribute to the development of plaque lesions by increasing inflammation.

Furthermore, the presence of microorganisms in atherosclerotic plaques is well recognized. Pathogenic microorganisms are able to infect arterial wall cells, facilitate local inflammation of the plaque, evoke an adaptive immune response and influence plaque progression. For example, *Chlamydia pneumoniae* infection enhances monocyte adhesion, induces foam cell formation, activates LDL receptors, and triggers inflammation and atherosclerosis (107). *Porphyromonas gingivalis* induces endothelial cell dysfunction, promotes VSMC proliferation, migration, and calcification and the formation of foam cells, which also leads to an imbalance of Tregs and Th cells, suppresses T cell immunity, facilitates inflammatory responses, and ultimately promotes the evolution of atherosclerotic plaques (108). Moreover, a study showed that gut microbiota (GM) are key environmental factors in regulating inflammation in atherosclerotic plaques (109). GM can influence the evolution of atherosclerotic plaques through direct invasion of plaques and by regulating cholesterol metabolism and the production of harmful metabolites. An animal study showed that transplantation of the pro-inflammatory GM from caspase1^-/-^ mice to *Ldlr^-/-^* mice enhanced systemic inflammation and increased atherosclerosis (110). In conclusion, microorganisms can regulate plaque progression by influencing inflammatory responses through multiple mechanisms.

In addition to bacteria, some viruses can also influence the evolution of atherosclerotic plaques. For example, cytomegalovirus (CMV) is capable of infecting almost all plaque immune cells, expressing viral cytokines and chemokines to promote an inflammatory environment, and triggering plaque formation through mechanisms such as induction of endothelial damage, increased lipid deposition, and VSMC proliferation and migration (111). *ApoE^-/-^* mice infected with CMV increases lesion size and IFN-γ levels, contributing to the development of atherosclerotic plaques (112). Other viruses including Epstein-Barr virus, herpes simplex virus type I, hepatitis virus, and human immunodeficiency virus have been reported to be associated with atherosclerosis, but the exact mechanisms need to be further studied. An in-depth study of the effects of pathogenic microorganisms on plaque evolution may also bring insights for the prevention and treatment of atherosclerosis.

## 5 Immune cell heterogeneity contributes to atherosclerotic plaque evolution

Leukocyte subsets accumulate at different stages of plaques and are involved in the immune response in the atherosclerotic lesion process. Single-cell RNA sequencing (scRNA-Seq) reveals heterogeneity of immune cells in atherosclerotic plaques (113). Studying immune cell heterogeneity could provide new insights into the evolutionary mechanisms of atherosclerotic plaques.

### 5.1 Monocyte/macrophage heterogeneity

**Figure 2:**
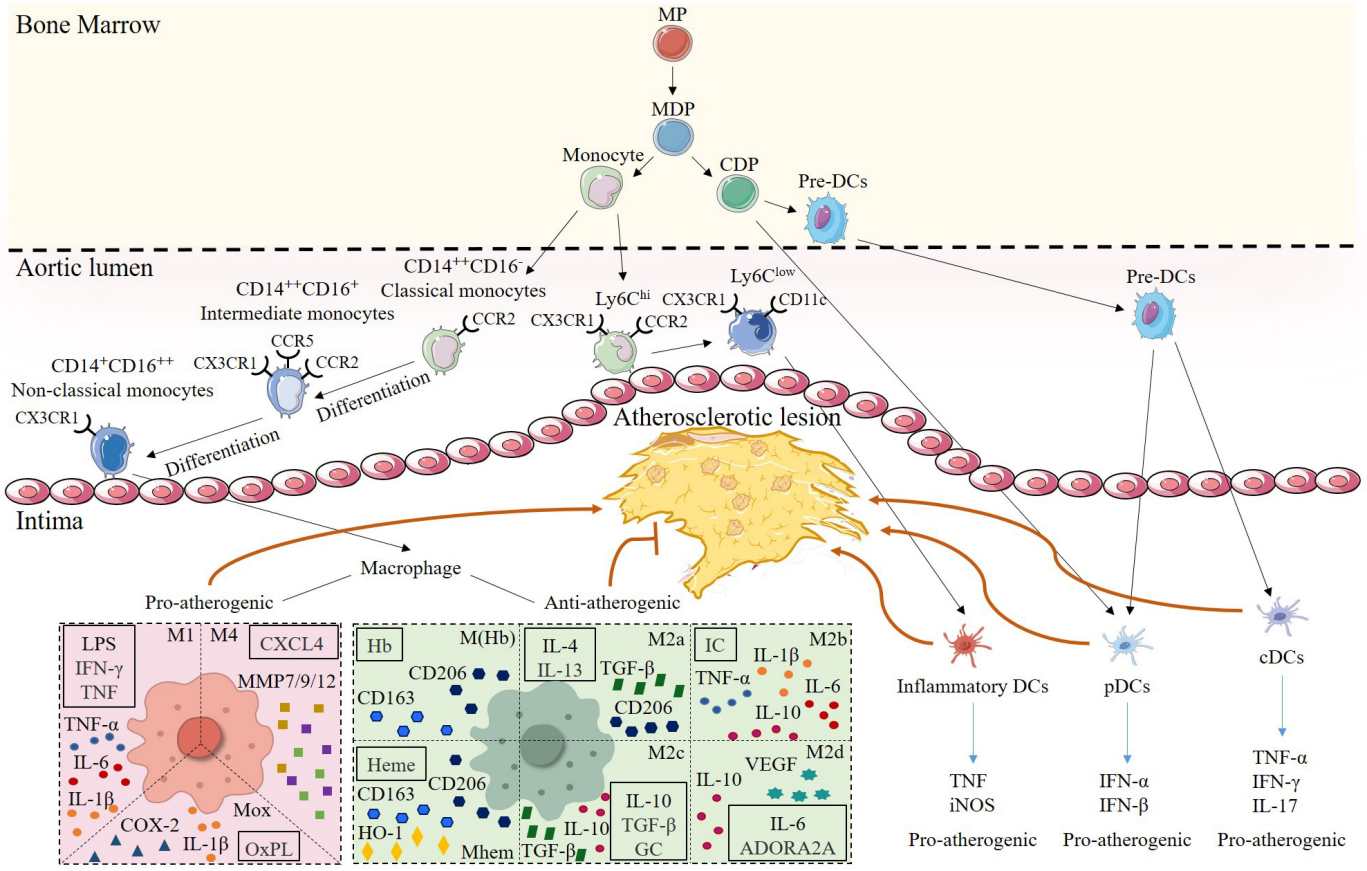
Heterogeneity of monocytes/macrophages and DCs in atherosclerotic lesions. Monocytes and DCs share a common origin in myeloid progenitor (MP). Monocyte-DC precursor (MDP) gives rise to monocytes and common DC precursor (CDP). Among them, mouse monocytes are classified into two subsets according to their surface expression of Ly-6C. Ly6C^hi^ subsets have pro-inflammatory functions and highly express CCR2. Ly6C^low^ subsets primarily patrol along the vascular endothelium, are engaged in tissue repair, and can express CD11c, and differentiate into inflammatory DCs to promote plaque progression in the plaque inflammatory microenvironment. Human monocytes are subdivided into three subsets based on the expression of CD14 and CD16.CD14^++^CD16^-^ classical monocytes, which express high levels of CCR2, can differentiate into CD14^++^CD16^+^ intermediate monocytes that express CCR5, and then further differentiate into CD14^+^CD16^++^ nonclassical monocytes, which are highly expressive of CX3CR1. Once monocytes enter the intima, they can mature into macrophages. Differentiation of different macrophage subsets depends on the stimulation of the inflammatory microenvironment within the lesion. Pro-atherogenic macrophages include M1, M4, and Mox, which are activated by Th1 cytokines, CXCL4, and OxPL, respectively, and secrete pro-inflammatory mediators, such as TNF-α, MMP, and IL-1β, to exert pro-inflammatory effects. Anti-atherogenic macrophages include M(Hb), Mhem, and M2. M(Hb) and Mhem are induced by Hb and Heme and secrete CD163 and CD206 to exert anti-inflammatory effects. M2 macrophages are subdivided into M2a, activated by IL-4 and IL-13; M2b, activated by IC; M2c, activated by IL-10, TGF-β and GC, and M2d, activated by IL-6 and ADORA2A. M2 macrophages mainly secrete Th2 cytokines such as IL-10 as well as TGF-β, which inhibit the development in atherosclerosis. CDP can further differentiate into pDCs and pre-DCs, and pre-DCs enter the arterial intima and become pDCs and cDCs, respectively, and play pro-atherogenic roles by secreting TNF or IFN.

Monocytes are heterogeneous populations and human blood monocytes are classified into three subpopulations according to differences in the expression of the lipopolysaccharide (LPS) receptor CD14 and the FcγIII receptor CD16: classical CD14^++^CD16^-^, intermediate CD14^++^CD16^+^ and non-classical CD14^+^CD16^++^ monocytes (114). In mice, monocytes are categorized into two major subpopulations depending on Ly-6C expression: Ly6C^hi^CCR2^+^CX_3_CR1^low^ and Ly6C^low^CCR2^-^CX_3_CR1^hi^ (115) (**Figure 2**). Similarly, macrophages are a heterogeneous cell population arising from the heterogeneity of monocytes and the stimulation of the inflammatory microenvironment of plaques. In recent years, the concept of macrophage heterogeneity has been better explored, but in atherosclerosis the traditional classification of M1 and M2 subsets continues to be used (116). A full understanding of the heterogeneous expression of plaque monocytes/macrophages will help to uncover the role of different functional phenotypes on plaque evolution.

Different monocyte/macrophage subsets play different roles in the evolution of atherosclerotic plaques. Classical CD14^++^CD16^-^monocytes account for about 85% of total monocytes, intermediate CD14^++^CD16^+^ monocytes for about 5% and non-classical CD14^+^CD16^++^ monocytes for about 10% (117). Classical monocytes, as major phagocytes, are capable of producing M1 macrophages and foam cells with strong peroxidase activity, producing ROS and secreting cytokines such as IL-10 in response to LPS and taking up LDL during infection and inflammation. They also exhibit high levels of CCR2 and CD62L, as well as low levels of CX3CR1. Human CD14^++^CD16^+^ intermediate monocytes are pro-inflammatory. This subset exhibits the highest ROS production and lowest peroxidase activity and secretes IL-1 and TNF-α in response to LPS associated with the evolution of atherosclerotic plaques. It also expresses CXCR1, CCR2, and CCR5 (118). It has been demonstrated that enzymatic degradation of LDL preferentially binds intermediate monocytes and is able to induce foam cell formation, suggesting that this subset has a pro-atherogenic effect (119). Non-classical monocytes are weak phagocytes that preferentially take up oxLDL and have an inflammatory effect, producing numerous inflammatory cytokines for example TNF-α and IL-1β. Additionally, they also express an elevated level of CX3CR1 and have a strong affinity for ECs, acting more like patrolling immune cells (120). Overall, human monocyte subsets differ not only in their proportions and phenotypes, but also significantly in their functions, but all promote plaque development.

Murine monocytes are the most studied. The Ly6C^hi^ subset resembles human classical monocytes and is referred to as ‘‘inflammatory’’ monocytes, while the patrolling Ly6C^low^ subset resembles non-classical monocytes in part and is referred to as ‘‘resident’’ monocytes (121). Ly6C^hi^CCR2^+^CX_3_CR1^low^ monocytes rely on CX3CR1, CCR2, and CCR5 for transport to plaques, while Ly6C^low^CCR2^-^CX_3_CR1^hi^ are recruited to plaques through CCR5. In the arterial intima, Ly6C^hi^ monocytes can differentiate into M1 macrophages, which go on to produce foam cells and produce inflammatory cytokines such as TNF-α, IL-6, ROS, and matrix metalloproteinases (MMPs) that actively contribute to plaque formation and progression (122). Research has demonstrated that the amount of Ly6C^hi^ cells in the blood increases dramatically as atherosclerosis progresses in hypercholesterolemic *ApoE^-/-^* mice, suggesting that Ly6C^hi^ monocytes have a crucial role in plaque progression (123). Ly6C^hi^ monocytes can be converted into Ly6C^low^ cells. Unlike Ly6C^hi^, Ly6C^low^ monocytes are recruited less to plaques but preferentially express the DC-associated marker CD11c (124). Ly6C^low^ monocytes rely on CX3CR1 to patrol healthy tissue in a long-range crawl across the endothelium, which allows them to rapidly invade infected tissue, initiate an early immune response and differentiate into macrophages when early inflammation occurs (125). Monocyte heterogeneity is important in the evolution of atherosclerotic plaques, but the mechanism of action of some subsets is unclear and much future research is needed.

Indeed, macrophage phenotype is influenced by microenvironmental factors within the atherosclerotic plaques. Both M1 and M2 macrophage populations are present and increasing in number throughout the atherosclerotic process (126). Monocytes are induced by GM-CSF and stimulated by Th1 cytokines, for example TNF-α and IFN-γ, to differentiate into M1 macrophages (127), which express various pro-inflammatory cytokines, including IL-13, IL-6, and TNF-α, leading to the recruitment of inflammatory cells and accelerating the development of plaques. In addition, M1 macrophages can also secrete MMPs, which degrade the ECM and cause plaque rupture. M1 macrophages accumulate in the rupture-prone shoulder area of the plaque, and both M1 and M2 macrophages are expressed at the fibrous cap near the necrotic core (126). Unlike M1 macrophages, M2 macrophages are induced to polarize by different cytokines like M-CSF, IL-4, IL-13, and IL-10 (127). Among these, large amounts of IL-13 are produced mainly from ILC2s.Activation of ILC2s has been shown to be associated with reduced atherosclerotic burden. Engelbertsen et al. (128) found that treating *Ldlr^-/-^rag1^-/-^* mice with IL-2/anti-IL-2 complexes resulted in the expansion of CD25^+^ ILC2s, which lowered very low-density lipoprotein cholesterol and atherosclerosis, and conversely ILC2s depletion led to an acceleration of the atherosclerotic process (129). Mantani et al. (130) also observed that IL-25 treatment of *ApoE^-/-^* mice inhibited the development of atherosclerosis by a massive expansion of ILC2s, an increase in IL-5 concentration, and greater numbers of B1 cells and IgM antibodies. Meanwhile, the expansion of ILC2s secreted large amounts of IL-13, which enhanced plaque stability and prevented the development of atherosclerotic lesions by increasing collagen deposition and promoting macrophage polarization towards M2 (131–133). According to the stimulation signal, M2 macrophages are further divided into four different subsets. M2a macrophages are induced by IL-4 and IL-13 to secrete anti-inflammatory cytokines including IL-10 and IL-1, which have strong effects on anti-inflammation, as well as high expression of mannose receptor, pro-fibrotic factors, and transforming growth factor involved in the repair of damaged tissues (134, 135). M2b macrophages can be stimulated by immune complexes, TLR agonists in combination with IL-1 receptor agonists, or LPS to produce and express large numbers of the anti-inflammatory cytokine IL-10, which exerts an immunomodulatory role in atherosclerotic plaques, but also secretes pro-inflammatory cytokines such as TNF-α, IL-1β, and IL-6 (121, 134). M2c macrophages are induced to activate by IL-10, TGF-β or glucocorticoids and they are capable of releasing IL-10 and pentraxin 3 for anti-inflammatory effects, and high expression of Mer receptor kinase for its efficient efferocytosis (136, 137). The process of M2d macrophage polarization is mediated by IL-6 and TLR stimulation of the adenosine A2A receptor, producing large amounts of IL-10 and vascular endothelial growth factor (VEGF) to promote angiogenesis and plaque growth in atherosclerotic plaques (138) (**Figure 2**). Overall, in addition to M2b macrophages, M2 macrophages produce many anti-inflammatory cytokines that maintain effective efferocytosis in atherosclerotic plaques as well as reducing plaque inflammation.

Recently, many research studies have revealed the presence of other macrophage subsets in atherosclerotic plaques, in addition to M1 and M2 types. M4 macrophages are induced by CXCL4, which is widely expressed in plaques, and exert atherosclerotic effects by producing MMP12, MMP7, and MMP9 leading to increased plaque vulnerability (139–141). The presence of M (Hb) and Mhem-type macrophages in areas of advanced atherosclerotic plaque bleeding is induced by hemoglobin and heme, respectively (142, 143). They characteristically express proteins and receptors such as the SR cysteine-rich type 1 protein M130 and macrophage mannose receptor 1 and activate related signaling pathways to increase liver X-receptor (LXR)-α activity to enhance cholesterol efflux, thereby preventing foam cell formation and exerting anti-atherogenic effects (143–145). Mox macrophages, which lack phagocytosis, account for approximately one-third of all macrophages in mouse advanced atherosclerotic plaques. Their formation is dependent on oxidized phospholipids and the transcription factor Nrf2, and they are capable of secreting pro-inflammatory cytokines such as IL-1β and cyclooxygenase 2, which contribute to plaque evolution and inflammation (146). It follows that macrophages can polarize in the complex microenvironment of the atherosclerotic plaque, and that the great heterogeneity of macrophages has a different impact on plaque evolution.

Single-cell techniques have greatly improved our understanding of macrophage subsets in plaques. Zernecke et al. (147) comprehensively analyzed leukocyte subpopulations identified by scRNA-Seq, and cell counting (CyTOF) and confirmed five macrophage subsets emerging in the aorta of atherosclerotic mice: resident -like macrophages, inflammatory macrophages, Trem2^hi^ foamy macrophages, interferon-inducible cell (IFNIC) macrophages and cavity macrophages. The newly discovered macrophage subsets exhibit heterogeneity in gene expression, with each subset having genes that are more significantly enriched in expression relative to the other subsets. Resident-like macrophages express *Lyve1*, *Timd4*, *Mrc1*, *Pf4* and *Ccr2 (* 148, 149). Inflammatory macrophages highly express the inflammatory chemokines *Ccl2-4* and *Cxcl2* and are also enriched in a large number of classical pro-inflammatory transcripts, such as *Il1α*, *Il1β*, and *Nlrp3*, which play an important role in regulating the progression of atherosclerosis (150). Trem2^hi^ foamy macrophages possess a gene expression profile that partly overlaps with those of inflammatory macrophages and in addition express *Lgals3*, an atherosclerosis biomarker that promotes the differentiation of monocytes to macrophages and is linked to alternative macrophage activation and plaque evolution (151, 152), as well as expressing the *Cd9* and *Ctsd*, the gene encoding cathepsin D. Cathepsin D is involved in the modification of LDL and facilitates phagocytosis of oxLDL by macrophages, which leads to the formation of foam cells (153). IFNIC macrophages are characterized by type I interferon and express a variety of interferon-inducible genes such as *Ifit3*, *Irf7* and *Isg15*, it is known that type I interferons usually have pro-atherogenic effects (154). Cavity macrophages express MHCII encoding genes as well as *Cd226*, *Itgax* and *Ccr2*, and the function of the cells is unknown (147). In conclusion, resident-like macrophages have anti-inflammatory properties, IFNIC macrophages and inflammatory macrophages are pro-inflammatory, Trem2^hi^ foam macrophages are not pro-inflammatory but function in lipid metabolism and cholesterol efflux (155, 156). Similarly, integrated analysis of scRNA-Seq data from immune cells in human atherosclerosis revealed that macrophages in human lesions exhibit similar transcriptional status to subsets of mouse aortic macrophages (157). The newly discovered subsets add an important dimension to our study of plaque macrophages, but the impact on plaque evolution and the specific mechanisms of action still need to be clarified by in-depth scientific studies and explorations.

### 5.2 DC heterogeneity

DCs are heterogeneous blood-borne professional antigen-presenting cells with the ability to capture, process, and present antigens to T cells that recognize antigens and induce antigen-specific immune responses (158). The functional heterogeneity of DCs identified in plaques suggests their complicated and multifaceted roles in atherosclerotic disease pathogenesis. The plaque inflammatory microenvironment and exogenous stimuli affect the DCs phenotype and are able to control the switch to an inflammatory or tolerant phenotype. DCs can be categorized into several subsets based on their origin, location, and function (159). In atherosclerotic plaques, the focus is on myeloid DCs (mDCs), plasmacytoid DCs (pDCs), pre-DCs, conventional DCs (cDCs), and inflammatory DCs (**Figure 2**).

mDCs are characterized by the expression of CD1c, CD11c, and CD33 and they secrete IL-12. In contrast the pDCs express CD123 and produce type I IFN (160). The differences in the surface markers of mDCs and pDCs results in inconsistent quantitative changes exhibited in atherosclerotic disease. For example, in coronary artery disease patients mDCs precursors, but not pDCs precursors, are significantly reduced, which is contrary to other research showing a marked reduction in the amount of pDCs and a remarkable increase in the amount of mDCs in patients with coronary artery disease (161, 162). In atherosclerosis, mDCs and pDCs were confined to the shoulder of lesions to produce IFN-α, demonstrating their association with plaque instability (163). mDC and pDC coexistence synergized TLR4 and TLR9 ligand inflammatory effects as demonstrated by increased production of TNF-α, IL-12, and MMP9 (164). The development and stereotyping of mDCs are modulated by a variety of transcriptional and hematopoietic growth factors, with *CCr7*, *Zbtb46*, and *Flt3* representing the core genes responsible for the development as well as functional and phenotypic maintenance of mDCs. mDCs have been shown to be associated with the pathogenesis of atherosclerosis. Various subsets of mDCs may have both atherogenic and atheroprotective activities during atherogenesis. The pro-inflammatory effects of mDCs include pro-inflammatory subsets that produce inflammatory molecules and initiate effector T cells. On the contrary, tolerogenic mDCs combat inflammation by inhibiting the activity of pro-inflammatory T cells and macrophages and by inducing immunosuppressive Tregs (165). Deficiency of FMS-like tyrosine kinase 3 ligand in mice leads to lack of CD103^+^CD11c^+^ mDCs and other DC subpopulations as well as advanced atherosclerosis. In *Ldlr^-/-^* mice, CD103^+^CD11c^+^ mDCs exert immunosuppressive effects through mechanisms that support FoxP3^+^ Treg homeostasis (166). These findings suggest that CD103^+^CD11c^+^ mDCs are atheroprotective. Transplantation of MyD88-deficient CD11c^+^ DCs into *Ldlr^-/-^* mice decreases stimulation of peripheral effector T cells, reduces the aggregation of effector T cells and Tregs in plaques, and results in an increase in plaque size owing to accumulation of myeloid-derived pro-inflammatory cells (167). Overall, mDC subsets play diverse roles in atherogenesis.

Several studies have discussed the role of pDCs in atherosclerotic plaque evolution. In a mouse model of atherosclerosis, depletion of pDCs using multiple antibodies against bone marrow stromal cell antigen 2 (BST2) promotes the accumulation of T cells in plaques, leading to enhanced atherosclerosis in *Ldlr^-/-^* mice (168); whereas in *ApoE^-/-^* mice, atherosclerosis is reduced by decreasing macrophage infiltration in plaques and stabilizing plaques by increasing collagen content (169, 170). The transcription factor E2-2/Tcf4 is an important regulator of pDCs development. Additional studies have found atheroprotective effects with specific deletion of the transcription factor E2-2/Tcf4 or impaired MHCII antigen presentation in pDCs, suggesting that in atherosclerosis, pDCs also promote MHCII-dependent antigen presentation through T-cell responses (171). Therefore, pDCs can drive immune responses in atherosclerosis and their function is influenced by environmental factors.

Pre-DCs are the final precursor stage in the formation of DCs, and they can develop into DCs without dendrites but require further development to obtain dendritic forms and full DC function. Little cell division is involved in their development into DCs, leading to different types of DCs being produced by different pre-DCs, such as some monocytes, pDCs, and cDCs (172). Some studies have reported increased accumulation of pre-DCs in plaques, which may be attributed to increased levels of circulating chemokines of CCL2, CCL5, and CXCL12 enhancing the entry of pre-DCs into plaques (173, 174). cDCs are also classified as migratory DCs, e.g., Langerhans cells and dermal DCs and lymphoid-tissue-resident DCs, which are not able to migrate to the lymph nodes. Recently, based on different developmental pathways, cDCs have been further classified into cDC type 1 (cDC1), including CD8α^+^ DCs in lymphoid tissues and CD103^+^ DCs in nonlymphoid tissues, and CD11b^+^ cDC2s (47). cDCs can be engaged in interactions with T cells and NKT cells, resulting in increased production of IFN-γ, IL-17, and TNF-α by T cells promoting plaque development. Among them, CD11b^+^ cDC2s were able to participate in lipid accumulation and foam cell formation (48). cDCs can regulate the evolution of atherosclerotic plaques in an antigen-dependent manner in part by modulating T cell activation and adaptive immune responses. Inflammatory DCs do not exist in a stable state, instead they appear as a result of inflammation (160, 172). CX3CR1 deficiency affects the accumulation of DCs in the aortic wall and significantly attenuates the atherosclerotic burden, which could demonstrate that inflammatory DCs may differentiate primarily from Ly6C^low^ monocytes and promote plaque evolution (175). The pro-inflammatory microenvironment at the atherosclerotic plaque provides an excellent option for the predominant differentiation of circulating DC precursors and monocytes toward inflammatory DCs, which in turn can promote the differentiation of naïve T cells into inflammatory and pro-atherosclerotic Th subsets, such as Th1 and Th17 (158). Inflammatory DCs act as an important player in the pro-inflammatory response at plaque lesion sites. In conclusion, pre-DCs develop into different subsets of mature DCs under the influence of different factors and function in the evolution of plaques.

### 5.3 T cell heterogeneity

In atherosclerotic plaques, T cells are less numerous than monocytes/macrophages, but like monocytes/macrophages that exhibit heterogeneity in markers and function, T cells also exhibit heterogeneity and regulate the adaptive immune response to plaque. CD4^+^ T cells are commonly found in atherosclerotic plaques, and CD4^+^ T cell subsets are substantially heterogeneous. In response to different stimulus activations, CD4^+^ T cells differentiate into different Th subsets, including Th1, Th2, Th17 and Tregs (**Table 1**).

**Table 1:** Heterogeneity of CD4^+^ T cell subpopulations.

CD4^+^ T cell subsets	Transcriptional regulators	Cytokines produced	Role in atherogenesis
Th1	T-bet	IFN-γ, TNF-α, TNF-β	Proatherogenic
Th2	GATA-3, c-Maf	IL-4, IL-5, IL-13	Atheroprotective/ Proatherogenic
Th17	RORγt, RORα, STAT3, AhR, RUNX1	IL-17	Atheroprotective/ Proatherogenic
Treg	FOXP3, RORγt	TNF-β, IL-10	Atheroprotective

Th1 is the prominent T cell subtype in atherosclerotic lesions and Th1 cytokines predominate in atherosclerotic mouse models and human plaques. They secrete mainly IFN-γ, which exerts a pro-atherogenic effect (176). IFN-γ is a major inducer of atherosclerotic lesions and promotes plaque development and instability in a number of ways, including endothelial dysfunction, recruitment of inflammatory cells, downregulation of intraplaque cellular cholesterol efflux, promotion of lipid deposition and foam cell formation, and induction of pro-inflammatory cytokine secretion (177, 178). IFN-γ^-/-^ mice crossed with *Ldlr^-/-^* mice showed significantly fewer atherosclerotic lesions compared to *Ldlr^-/-^* mice after 8 weeks of feeding a cholesterol diet (179). Similarly, *ApoE^-/-^* and IFN-γ receptor double knockout mice showed a substantial reduction in atherosclerotic lesions, as evidenced by reduced lipid accumulation and reduced numbers of inflammatory cells, compared to *ApoE^-/-^* mice after 3 months of feeding a Western type diet (180), whereas exogenous injection of IFN-γ to *ApoE^-/-^* mice resulted in increased lesions (181). All the above studies suggest that IFN-γ plays a facilitating role in the evolution of plaques. T-bet is a transcription factor essential for Th1 cell differentiation, and *Ldlr^-/-^* mice lacking T-bet show diminished atherosclerosis (182). This further suggests that Th1 cells promote atherogenesis and also confirms that atherosclerosis is driven by the Th1 response.

Th2 cells are poorly represented in atherosclerotic plaques, and their function in atherosclerosis is still controversial. Th2 cells typically produce cytokines such as IL-4, IL-5, and IL-13. IL-4 can effectively inhibit the Th1 response, Th1 cytokine formation and IFN-γ secretion, and has atheroprotective effects (176, 183). However, other studies have shown that IL-4 deficiency leads to decreased atherosclerotic lesion formation in *Ldlr^-/-^* mice, suggesting a pro-atherogenic effect of Th2 cells (184). Furthermore, in angiotensin II-induced atherosclerotic *ApoE^-/-^* mice, IL-4 administration showed no effect on plaque development (185). In summary, IL-4 exerts different effects on the development of atherosclerotic plaques. Unlike IL-4, IL-5 and IL-13 have a defined atheroprotective effect. IL-5 induces B1 cells to produce atheroprotective natural IgM antibodies specific for oxLDL. Second, IL-5 deficiency enhances plaque formation (72). Similarly, plaque development is accelerated by IL-13 deficiency, and IL-13 exerts atheroprotective effects by increasing collagen content, reducing monocyte recruitment, and inducing M2-type macrophage polarization (133). Furthermore, ILC2 cells, which are thought to correspond to Th2 cells, have been found to perform class II MHC restricted antigen presentation and to orchestrate Th2-like immune responses (186). IL-25 as well as IL-33 induced by Th2 cells can promote the proliferation of ILC2 cells, and the activation of ILC2 cells in turn promotes the Th2 response, thereby reducing the burden of atherosclerosis (187). In conclusion, the exact role of Th2 cells in atherosclerosis is still unclear. Possible reasons for this include different research teams have various experimental designs, study methods, and focuses; cytokines produced by Th2 cells, especially IL-4, exhibit complex biological properties; most studies have been conducted in animal models, but animal models of atherosclerosis have certain limitations; and there are also complex interactions between immune cells, and Th2 may synergize with or antagonize other immune cells, thereby affecting its role in atherosclerosis. More in-depth and comprehensive studies are needed to elucidate the role of Th2 in atherosclerosis.

The Th17 subset has been identified in atherosclerosis-prone mouse and human atherosclerotic plaques and its role remains controversial (188). Differentiation of Th17 cells is mediated by retinoid-related orphan nuclear receptor (ROR)γt (189) and other transcription factors such as RORα, STAT3 (190), Aryl hydrocarbon receptor, and runt-related transcription factor 1 (191, 192). Unlike classical Th1 and Th2 cells, Th17 cells exhibit significant heterogeneity due to the unstable expression of RORγt and its property of being influenced by environmental cues. Th17 cells mainly secrete the cytokine IL-17. The effect of IL-17 in atherosclerotic plaques would seem to be complex. IL-17 further exerts pro-atherosclerotic effects by inducing IL-6, CXCL8 and CXCL10, and GM-CSF in VSMCs to promote inflammatory activation (193). On the other hand, the atheroprotective effect of IL-17 is associated with inhibition of the pro-atherogenic factor IFN-γ and production of the anti-atherogenic factor IL-10, as well as suppression of the expression of vascular cell adhesion molecule 1 (VCAM-1) leading to a reduction in leukocyte recruitment within the lesion (188). Due to the complexity of IL-17’s action, several related experimental studies have yielded different results. Experiments with functional blockade of IL-17 have demonstrated that IL-17 has pro-atherosclerotic effects, and that inhibition of IL-17 reduces plaque development, decreases plaque vulnerability, and reduces leukocyte infiltration (194). Another study showed that IL-17 exerts atheroprotective effects by promoting collagen cap formation and stabilizing plaques (195). In conclusion, Th17 plays a complex role in atherosclerosis, as demonstrated by its ability to both promote atherosclerosis and inhibit its development.

Tregs are a highly heterogeneous population of CD4^+^ T cells with immunosuppressive functions. In the pro-inflammatory microenvironment formed by pro-inflammatory factors such as IL-1β, IL-2, and IL-23, CD4^+^FOXP3^+^ T cells co-express FOXP3 and RORγt transcription factors and are able to produce IL-17 upon stimulation (196). IL-1β and IL-2 induce the differentiation of the initial FOXP3^+^ Tregs into Th17 cells, and similarly IL-23 is able to differentiate Tregs into Th17 cells through inducing a high level of RORγt expression and enhancing the loss of FOXP3 (197, 198). Tregs have been clearly demonstrated to be atheroprotective, with reduced numbers and impaired suppressive function linked to the evolution of atherosclerotic plaques (199). The amount of Tregs was reduced in mice that developed atherosclerosis compared to *ApoE^-/-^* mice in which no atherosclerotic plaques were detected (200). In a functional study of CD4^+^FOXP3^+^ Tregs, it was found that when Tregs were depleted by anti-CD25 antibodies, plaque vulnerability increased, and lesion progression was accelerated (201). Despite the small number of CD4^+^FOXP3^+^ Tregs found in mouse and human plaques, their secretion of cytokines, such as TGF-β and IL-10, has a profound impact on plaque evolution. In *ApoE^-/-^* mice, specific blockade of TGF-β signaling promotes atherosclerosis, as evidenced by increased macrophage activation, reduced collagen, increased plaque size, and increasing instability. In contrast, TGF-β overexpression reduced plaque vulnerability and atherosclerosis (202). Similarly, IL-10-deficient mice were found to have a substantially increased susceptibility to atherosclerosis compared to normal mice, as evidenced by increased T cell infiltration, high IFN-γ expression, and reduced collagen content (203). Overall, Tregs exert atheroprotective effects on atherosclerosis by secreting cytokines to inhibit plaque-associated inflammatory responses.

Using scRNA-seq screening methods, Winkels et al. (204) found five T cell populations in *ApoE^-/-^* mice; Gu et al. (205) identified three T cell populations; and Cochain et al. (150) detected four T cell populations in *Ldlr^-/-^* mice. Recently, Winkels et al. (206) synthesised most of the available scRNA-seq studies and outlined four T cell phenotypes that have been more consistently studied: *Cxcr6*-expressing T cells, where CXCR6 is expressed by CD4^+^ T cells and NKT cells and is able to direct T cell homing. The overall lack of CXCR6 reduces atherosclerosis and T cell accumulation (52). Naive T cells, expressing *Cd28^+^Ccr7^+^*, increase IL-7 signaling and support T cell survival, differentiation as well as proliferation in more advanced atherosclerosis (207). *Cd8^+^* cytotoxic T cells, in scRNA-seq, about 31% of the T cells were identified as cytotoxic T cells based on the genotype and diet of the tested mice. The cells mainly expressed *Cd8a/b*,*Nkg7*, *Ms4a4b*, *Ccl5* and *Gzmk*. Depletion of CD8^+^ T cells promotes atherosclerosis and reduces plaque stability (204). Another population of T cells that express both CD4 and CD8 or neither may represent thymocyte-like T cells (204), but this is still controversial.

### 5.4 B cell heterogeneity

B cells are heterogeneous populations of lymphocytes derived from bone marrow and composed of multiple subsets of cells with distinct localization properties, activation requirements, survival characteristics, and immunoglobulin secretion profiles (64). In mouse atherosclerosis, B cells are subdivided into two main subsets, B1 and B2, where B1 cells are divided into CD5^+^ B1a cells and CD5^-^ B1b cells according to the expression of the leukocyte differentiation antigen CD5, and B2 cells contain follicular (FO) and marginal zone (MZ) B cells. CyTOF, and scRNA-Seq also confirmed the presence of B1- and B2-like cells in mouse atherosclerotic vessels (204).B1-like cell cluster is enriched for B1 cell genes (*Tppp3*, *S100a6*, and *Cd9*), and the B2-like cell cluster shares gene expression with germinal center and MZ B cells, for example, *Fcer2a* and *Cd23 (* 147).Various subsets of B cells can contribute to diverse, and sometimes contradictory roles in the development of atherosclerotic plaques.

B1 cells are intrinsic immune cells that are usually found in plasma membrane cavities such as the pleural and peritoneal cavities. Without the need for Th cells, both B1a and B1b cells can secrete IgM under antigenic stimulation, which specifically binds to LDL oxidizing epitopes in atherosclerotic plaques and reduces lipid uptake by macrophages, thus decreases the formation of foam cells. At the same time, IgM specifically binds to apoptotic cells so that inflammatory cells can be eliminated, thus slowing down atherosclerosis progression (64). B1a cells undergo migration to the spleen and differentiate into GM-CSF-producing innate response activator (IRA) B cells stimulated by LPS. In atherosclerotic mice, IRA-B cells congregate in large numbers in secondary lymphoid tissues, activate the Th1 immune response, stimulate extraparenchymal hematopoiesis, and activate DCs to promote atherosclerotic plaque formation (208). Kyaw et al. (209) found that splenectomy in *ApoE^-/-^* mice resulted in a decrease in peritoneal B1a cells as well as a substantial reduction in plasma IgM levels; however, transfer of B1a cells to splenectomized mice was effective in attenuating atherosclerotic lesions and reducing plaque necrotic cores and apoptotic cells. However, the role of B1b cells in atherosclerosis remains uncertain and necessitates additional investigation. Overall, B1 cells exert a protective effect against atherosclerosis primarily through the secretion of natural IgM antibodies that bind oxLDL and apoptotic cells.

B2 cells are what we commonly refer to as mature B lymphocytes, produced in the bone marrow and differentiated in secondary lymphoid tissues. Preliminary studies on the role of B2 cells in atherosclerosis have demonstrated their pro-atherogenic effect. The use of a CD20-specific monoclonal antibody to selectively remove B2 cells, but not B1a cells, from *ApoE^-/-^* and *Ldlr^-/-^* mice reduced atherogenesis and progression of atherosclerosis (73, 74). In addition, the fact that B2 cell depletion was correlated with the reduction of activated splenic CD4^+^ T cells, T cells proliferation, and diseased T cells suggests that B2 cells through a T cell-dependent mechanism exacerbate atherosclerosis (210). Consistent with the effects of B2 cell depletion, Kyaw et al. (74) found that the introduction of splenic B2 cells, but not B1 cells into lymphocyte-deficient recombinase activating gene 2 (*Rag2*)*^-/-^γ-chain^-/-^ApoE^-/-^* or B-cell-deficient/*ApoE^-/-^* mice through adoptive transfer exacerbated atherosclerosis. The pro-atherogenic effect of B2 cells was further demonstrated by studies of B cell-activating factor receptor (BAFFR)-deficient effects in atherosclerosis-prone mice. Being a member of the tumor necrosis factor receptor family, BAFFR plays a crucial role in the maintenance of mature B2 cells (211). In a separate study, Kyaw et al. (212) showed that selective inhibition of BAFFR with an anti-BAFFR antibody resulted in depletion of B2 lymphocytes and reduction of atherosclerosis in *ApoE^-/-^* mice. Collectively, these studies provide clear evidence that substantial reduction in B2 cell count mitigates the progression of atherosclerosis. In contrast to Kyaw et al., however, Doran et al. (213) discovered that adoptive transfer of *ApoE^-/-^* mouse splenic B2 cells into cholesterol-fed μMT/*ApoE^-/-^* mice significantly reduced the area of atherosclerotic plaques in mice. This discrepancy may be due to differences in the ratio of FO to MZ B cells or in the genetic background of the mice. Further research has confirmed the specific role of FO and MZ B2 cell in atherosclerosis. Namely, FO B cells promote atherosclerosis primarily through IgG production and activation of Th1 cells (214). Similarly, MZ B cells promote atherosclerotic plaque progression by secreting IgG, activating follicular helper T (Tfh) cells, and inducing the expression of inflammatory factors such as IFN-γ (215).

The variety of functions performed by B cells, such as antibody production, cytokine release, and antigen presentation, as well as the unique ways in which B cells adapt to the inflammatory microenvironment of plaques, have led to different B cell subsets exhibiting complex heterogeneity in the evolution of atherosclerotic plaques.

## 6 Immunotherapy for atherosclerosis

The evolution of atherosclerotic plaques is regulated by both the immune system and inflammatory responses, and the study of immune as well as anti-inflammatory based therapies for atherosclerosis is of great clinical importance. Canakinumab is a human monoclonal antibody (mAb) that binds to IL-1β and blocks the interplay of IL-1β with IL-1R, preventing the inflammatory response from occurring. IL-1β plays multiple roles in the atherosclerotic process, including promoting adhesion of immune cells to vascular endothelial cells, promoting proliferation of VSMCs, and inducing procoagulant activity (216). A large-scale, double blind, randomized clinical trial, the Canakinumab Anti-Inflammatory Thrombosis Outcome Study (CANTOS), has confirmed for the first time the inflammatory hypothesis of atherosclerosis (217). By dividing 10,061 atherosclerotic patients with prior myocardial infarction and a high-sensitivity C-reactive protein (hs-CRP) ≥2 mg/L in 39 countries into a placebo group and three different doses of canakinumab, administered subcutaneously every three months. The risk of canakinumab on primary endpoint events including nonfatal myocardial infarction, nonfatal stroke, and cardiovascular death was assessed. Ultimately, the 150 mg and 300 mg doses of canakinumab were found to reduce patients’ levels of hs-CRP and other inflammatory markers, the risk of cardiovascular events, and the incidence and mortality of selected cancers (218). It is worth noting that the use of canakinumab is accompanied by certain risks, and further studies are needed to investigate its precise mechanism of action as well as its safety and efficacy in different patients. Overall, the CANTOS study opens up new avenues for immunotherapy in cardiovascular disease.

Advances have also been made in the study of antibody immunotherapies targeting other cytokines that play pro-inflammatory roles in plaque evolution, as well as chemokines and their receptors that also have important roles. In patients with psoriatic arthritis, the use of anti-TNF-α antibodies significantly reduced the size of carotid plaques and inhibited plaque development (219). Additionally, adalimumab treatment steadily reduced the levels of E-selectin, hs-CRP, and IL-22, suppressed systemic inflammation, and decreased the risk of atherosclerosis (220). At chronic kidney disease patients, the anti-IL-6 antibody remarkably decreased biomarkers of atherosclerotic inflammation and thrombosis (221). Blocking the binding of CCR2 to its ligand CCL2 with the highly specific MLN1202 in patients with atherosclerotic cardiovascular risk reduced the levels of hs-CRP (222).

Anti-programmed cell death protein 1 (PD-1) mAb has recently been found to exhibit potential therapeutic efficacy in the treatment of atherosclerosis. There are activated subsets of PD-1^+^ T cells in the plaques that act as pro-inflammatory (223). PD-1 belongs to the immunoglobulin superfamily and is mainly expressed on the surface of T cells. It usually binds to its ligands PD-L1 and PD-L2, mediating immune suppression signals. It plays an important role in regulating T cell functions such as proliferation, survival, cytokine production and other effector functions and maintaining immune system homeostasis (224). A latest study by Fan et al. (225) found that the use of an anti-PD-1 mAb that binds to Fc was effective in reducing the size of human atherosclerotic plaques. The potential mechanism of action is that the Fc-binding ability of the anti-PD-1 mAb enables it to be trapped by the Fcγ receptors, which then interact with PD-1 expressed on the surface of T cells as an alternative PD-1 ligand to inhibit PD-1^+^ T-cell function in atherosclerotic plaques. This finding suggests that T cell- targeting immunotherapy could be a novel strategy to address atherosclerosis. Here we have only reviewed relevant antibody immunotherapies that have been used in human clinical trial studies (**Table 2**). It is known that quite a number of immune molecules show considerable therapeutic prospects in animal models. We believe that future studies will reveal more targets and translate them into clinical applications, providing more options and possibilities for the treatment of atherosclerosis.

**Table 2:** Antibody immunotherapy for human atherosclerosis.

Antibody Type	Study	Mechanism	Effect on atherosclerosis	Reference
Anti-IL-1β (e.g. canakinumab)	Atherosclerotic patients (prior myocardial infarction and hs-CRP ≥2 mg/L)	Block the interaction of IL-1β with IL-1R; Reduce hs-CRP and IL-6 levels	Reduce atherosclerosis; Decrease risk of primary endpoint events	(218)
Anti-TNF-α (e.g. adalimumab)	Psoriasis patients (atherosclerotic plaques)	Suppress systemic inflammation	Inhibit plaque progression; Lower risk of atherosclerosis	(219, 220)
Anti-IL-6 (e.g. ziltivekimab)	Chronic kidney disease patients (hs-CRP ≥ 2 mg /L)	Reduce hs-CRP levels	Reduce atherosclerotic inflammation; Reduce thrombosis	(221)
CCR2 mAb (e.g. MLN1202)	Patients at high risk for ACD (≥2 risk factors and hs-CRP >3 mg/L)	Block the binding of CCR2 to its ligand CCL2;Reduce serum CRP levels	Decrease the risk of ACD	(222)
Anti-PD-1 mAb	Tumor patients (atherosclerotic plaques)	Inhibit the function of PD-1**^+^** T cells	Reduce atherosclerotic plaque size	(225)

Antigen-antibody reactions play a pivotal role in the evolution of plaques. Therefore, the use of immunomodulatory strategies to activate immune responses against relevant antigens has the potential to alter the natural course of atherosclerosis. The purpose of vaccination is to prevent the progression of atherosclerosis by stimulating the body to produce antibodies that block the target antigen. What has received earlier attention from researchers is LDL-related vaccines. OxLDL is thought to be critical in causing intimal inflammation and foam cell formation in atherosclerosis. One of the most studied is the specific malondialdehyde-modified apolipoprotein B100 (MDA-ApoB100), which is a relatively important oligopeptide fragment of oxLDL molecules with strong antigenicity (226). Fredrikson et al. (227) reported that *ApoE^-/-^* atherosclerotic mice vaccinated with a peptide vaccine of the MDA-ApoB100 fragment showed a reduction of atherosclerotic plaques by up to 60%, and a significant increase in collagen content in the residual plaques, and a decrease in macrophages, which increased the stability of plaques to some extent and inhibited plaque evolution. In addition to more studies on oxLDL-related vaccines, the results of cholesterol transporter protein (CETP) vaccine studies have been promising. The primary function of CETP in atherosclerosis is to regulate the transport of high-density lipoprotein (HDL) cholesterol to LDL cholesterol (228). CETP-associated vaccines play a role in inhibiting plaque development through mechanisms such as elevating HDL levels, decreasing LDL levels to promote reverse cholesterol transport, and reducing plaque burden (229). Other vaccines such as CD40L and PCSK9 have been found to play an inhibitory role in atherosclerotic plaque evolution (230–233). Plaque evolution is a complex, multifactorial process. From the perspective of developing an anti-atherosclerotic vaccine, not only can we explore the impact of immunotherapy on the mechanism of plaque evolution, but also contribute substantially to the prevention and treatment of atherosclerosis.

Inflammation is a well-studied therapeutic target, and strategies to control inflammation have been successfully applied against many diseases. After many studies it appears that inflammation has also emerged as an important target for the treatment of atherosclerosis. Anti-inflammatory therapeutic agents for atherosclerosis have been extensively studied in recent years. Among the most widely researched and used drugs are statins, which are inhibitors of 3-hydroxy-3-methylglutaryl coenzyme A reductase and are usually used to treat atherosclerosis. They are the drugs of choice for clinical lipid-lowering, with commonly used drugs such as rosuvar, atorval, and simvastatin calcium tablets. Statins exert various anti-inflammatory effects in addition to their lipid-lowering properties. Statins can inhibit macrophage growth, reduce the level of pro-inflammatory cytokines, downregulate the expression of adhesion molecules and chemokines, and prevent monocyte recruitment (234), as well as inhibit T cell activation and attenuate inflammatory responses through direct binding to lymphocyte function-associated antigen-1 (235). Furthermore, statins promote the stabilization of plaques by decreasing MMP expression and inhibiting tissue factor to reduce the risk of thrombotic events (236). In summary, statins can prevent, reduce, and even reverse atherosclerotic plaque burden. Reducing inflammation may be a key mechanism by which statins alter plaque biology and slow disease progression.

In addition to statins, there are several common anti-inflammatory drugs that are also used to treat atherosclerosis. Colchicine is utilized for treating inflammatory diseases such as atherosclerosis due to its anti-inflammatory and antifibrotic activity. Its anti-inflammatory mechanism is mainly through downregulation of multiple inflammatory pathways including inhibition of phospholipase A2, reduction of leukotriene B4 and prostaglandin E2 release from monocytes, inhibition of neutrophil function through microtubule proteins, and reduction of endothelial adhesion, which leads to inhibition of inflammation and increase of plaque stabilization (237–239). Methotrexate is a better non-specific anti-inflammatory agent for treating atherosclerosis due to its lesser effect on the levels of atherosclerosis-related cytokines. Methotrexate performs its anti-inflammatory effect by inhibiting folate metabolism and reducing T-cell proliferation (240). In addition, methotrexate can regulate the expression of intercellular cell adhesion molecule-1, E-selectin, and VCAM-1, suppress cyclooxygenase and lipoxygenase, decrease C-reactive protein levels, and regulate the secretion of IL-6, TNF-α, and metalloproteinases, thereby regulating plaque evolution (241). In summary, targeting inflammatory pathways to prevent and treat atherosclerosis is a promising new avenue.

## 7 Conclusion

Atherosclerosis is defined as an inflammatory disease, with inflammatory responses throughout the disease progression, and inflammatory mediators, cytokines, through inflammatory signaling pathways exerting different roles in the regulation of the plaque inflammatory microenvironment. Many studies have clearly demonstrated that immune responses mediate the entire process of atherosclerotic plaque evolution, including initiation, progression, and thrombotic complications. Crosstalk between innate and adaptive immune pathways strongly regulates plaque activity and progression, while the heterogeneity of immune cells plays a pivotal role in plaque evolution.

The evolution of atherosclerosis involves complex interactions of immune cells as well as phenotypic plasticity. Omics studies, especially scRNA-seq, have highlighted the specific transcriptional profiles of various cell lineages at the site of atherosclerotic lesions. The detailed map of the cells revealed not only enhances our understanding of the heterogeneity of different cells in the plaque environment, but also deepens our comprehension of the mechanisms of disease occurrence and progression and provides a solid scientific basis for the development of novel precision therapeutic strategies. By combining high-throughput omics data with experimental studies to analyze the gene expression profiles and functional properties of specific cell subsets, it is possible to develop targeted drugs against specific cell subsets or the immune molecules they produce, such as immune checkpoint inhibitors against PD1^+^ T cells (225), antibodies against pro-inflammatory cytokines or chemokines (218–222). Gene expression products and functional markers may also become important biomarkers for assessing atherosclerosis disease, predicting progression and monitoring treatment effects, and the development of these biomarkers could help to achieve early diagnosis and precise treatment of the disease. Furthermore, adjusting the dose and type of therapeutic drugs according to the number of immune cells and the level of cytokines produced, and developing a more personalized treatment plan can improve the effectiveness of treatment and reduce adverse reactions. In addition, understanding the interactions between different cell subsets and exploring the possibility of combining drugs, such as an integrated treatment strategy combining lipid-lowering and anti-inflammatory drugs, has shown certain efficacy in clinical trials (242, 243), with a view to achieving breakthroughs in reducing plaque formation, stabilizing lesions and preventing MACEs.

However, the vast majority of investigations into the pathophysiologic mechanisms of atherosclerotic plaque pathology have originated from experimental animal models, and there are significant barriers to translating interventions from animal models to the clinic, primarily because mouse models reproduce human atherosclerosis to a very limited extent. It is encouraging that the integration of bioinformatics-, transcriptomics-, and proteomics-based datasets will help us to define the immune profile of human atherosclerosis in a more comprehensive manner. As multidimensional approaches continue to progress, it is expected that the cellular and molecular mediators associated with human atherosclerotic plaques will be characterized in depth, and new potential therapeutic targets will undoubtedly emerge. It is our firm belief that our understanding of immune-mediated processes in atherosclerosis will continue to evolve, and the specific mechanisms of atherosclerotic plaque evolution will be further resolved from an immunologic perspective.
